# Progress in Electrochemical and Fluorescence Sensors for Propyl Gallate Monitoring in Food Samples

**DOI:** 10.3390/bios16020070

**Published:** 2026-01-24

**Authors:** Khursheed Ahmad, Sanjeevamuthu Suganthi, Chellakannu Rajkumar, Shanmugam Vignesh, Rohit Kumar Singh Gautam, Tae Hwan Oh

**Affiliations:** 1 School of Chemical Engineering, Yeungnam University, 280 Daehak-Ro, Gyeongsan 38541, Republic of Korea; 2 Department of Mechanical Engineering, Teerthanker Mahaveer University, Moradabad 244001, India; drrohit.engineering@tmu.ac.in

**Keywords:** propyl gallate, food additive, electrochemical sensors, antioxidants, fluorescence

## Abstract

Recent years have witnessed significant growth in the development of propyl gallate (PG) sensors. PG can be monitored by various approaches, such as electrochemical and fluorescence methods. The electrochemical approaches have several advantages, such as low cost, a benign fabrication process, and high sensitivity and selectivity. Similarly, the fluorescence method has its own advantages, including low cost, high sensitivity, and fast response. Both methods are promising approaches for the monitoring of PG compared to chromatographic methods. In this mini-review article, we review the progress in the preparation of materials for the determination of PG using electrochemical and fluorescence methods. The fabrication of electrodes and the working principle for PG detection are illustrated. The challenges and future perspectives for PG detection are discussed.

## 1. Introduction

In today’s world, while access to fresh food direct from farms has become easier, ensuring its freshness, safety, and suitability for long-term storage has become increasingly important [1]. The use of antioxidants, food additives, and preservatives is a widely used approach to protect/store food for long term uses [2]. Antioxidants, particularly propyl gallate (PG), a phenolic antioxidant, are extensively used in the food industry to enhance the shelf-life of fats, mayonnaise, noodles, edible oils, etc. [3,4]. PG hinders degradation, rotting, and changes in food color, owing to its antioxidative properties. Despite its potential applications in various fields, PG has several negative effects on human beings and the environment [5]. Residues of PG are released from the food industry and pharmaceutical industries via wastewater streams [6]. Due to the low biodegradability of PG, ecological systems are being compromised [7]. High concentrations of PG used as a preservative in foods may reduce their nutritional value, and PG may also exhibit harmful effects on human health due to its chemical properties [8]. Excessive intake of PG may also cause damage to the liver and kidneys; therefore, accurate determination of PG levels is of particular importance. Conventional methods such as chromatography and capillary electrophoresis are sensitive techniques, but they have their own limitations for food quality monitoring applications [9,10]. The conventional methods are time-consuming for the determination of the targeted analytes. Moreover, portability issues and the requirement of highly trained operators remain significant challenges. Therefore, there is a need to develop or explore simple, cost-effective, and portable techniques for the determination of PG in food samples.

**Figure 1:**
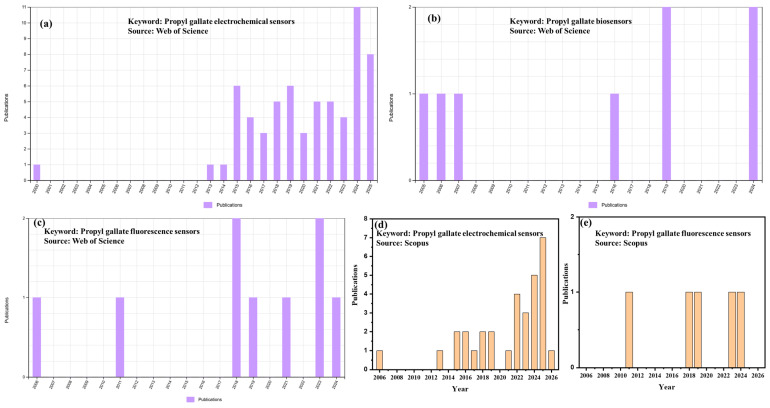
Bar graph showing reported sensors for PG detection. Keywords (Web of Science): (**a**) Propyl gallate electrochemical sensors, (**b**) biosensors, and (**c**) fluorescence sensors (source = Web of Science). Keywords (Scopus): (**d**) Propyl gallate electrochemical sensors and (**e**) fluorescence sensors.

Recently, electrochemical sensors and biosensors have received enormous interest because of their excellent selectivity, sensitivity, repeatability, reproducibility, cost-effectiveness, and simple fabrication procedures [11,12,13]. The performance of the sensors and biosensors is greatly affected by the physicochemical properties of the sensing materials. Given this, nanostructured and conductive materials are used as sensing materials for the monitoring of antioxidants in food samples [14,15]. Carbon-based materials such as carbon nanotubes (CNTs) [16,17] offer several advantages, such as high surface area, porosity, and charge transport properties that may enhance the electrochemical reactions at the surface of the working electrode. Carbon-based materials also enhance the conductivity of the low-conducting metal oxides. Thus, it is expected that carbon-based materials and their hybrids could be useful electrode materials for the construction of PG electrochemical sensors. Metal oxides and hybrid composites are also promising materials for the development of electrochemical sensors. However, as per the Web of Science and Scopus reports, only a limited number of sensors are available for the determination of PG, shown in Figure 1a–e.

On the other hand, the fluorescence method has also gained significant attention due to its high sensitivity, nondestructive measurement, and rapid response for the monitoring of the targeted analyte [18,19,20]. As per the observations, it was found that the electrochemical method has several advantages for the determination of PG, but the sensing performance of the fluorescence method cannot be neglected. Therefore, research on the monitoring of PG in food samples is ongoing for both methods. To date, only a few reports are available on electrochemical sensing and fluorescence-based detection of PG, as represented in Figure 1c,e. Thus, combining advances in PG monitoring using electrochemical and fluorescence methods would be valuable for researchers developing PG sensors. This mini-review article summarizes the progress in the monitoring of PG using electrochemical and fluorescence technologies. The challenges and current perspectives for PG detection are discussed in the Conclusions Section.

## 2. Electrochemical Sensors

**Scheme 1:**
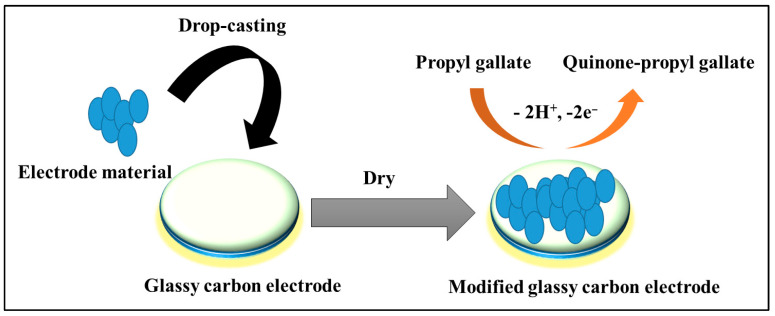
Schematic representation of the electrochemical sensor for PG sensing.

Before discussing the progress in electrode materials for PG detection, it is worth briefly describing the role of the electrode and the fabrication process in the determination of PG. The electrochemical sensors need the fabrication/modification of working electrodes for the monitoring of PG in food samples. Generally, glassy carbon electrodes (GCEs) are widely used for the fabrication of PG electrochemical sensors. The drop-casting approach is one of the simplest and most cost-effective techniques for the construction of electrochemical sensors. The electro-catalyst ink can be drop-cast onto the active area of the GCE and dried in ambient conditions for a few hours. The schematic representation of the construction of a PG electrochemical sensor is shown in Scheme 1.

### 2.1. Carbon-Based Materials

It is well-understood that carbon-based materials are promising electrode materials for the development of electrochemical sensors due to their high conductivity, porous nature, larger specific surface area, and charge transport properties. Thus, multi-walled CNTs (MWCNTs) were explored as an electro-catalyst for the determination of PG [21]. In connection with this, a gold (Au) electrode was modified with MWCNTs to fabricate the PG sensor. This fabricated electrode exhibited reasonable catalytic properties that may be beneficial for electrochemical sensing applications. In addition, authors also evaluated the electrochemical active surface area (ECSA) of the modified electrode by employing the Randles–Sevick equation as given below,

I_p_ = 2.69 × 10^5^A*_n_*^3/2^D_R_^1/2^C*v*^1/2^ (1)

(where I_p_ = peak current, *n* = number of electrons transferred, A = surface area of the electrode, D_R_ = diffusion coefficient (7.6 × 10^−6^ cm^2^s^−1^), C = concentration of the redox analyte, and *v* = scan rate).

The ECSA of the MWCNT-modified Au electrode was found to be 1.9932 cm^2,^ which was higher compared to the bare Au electrode (0.9842 cm^2^). Thus, it can be noted that the high ECSA value of the MWCNT-modified Au electrode may enhance the oxidation of PG. The authors used differential pulse voltammetry (DPV) for the quantification of PG, and the obtained results demonstrated a detection limit (DL) of 0.63 µM. The DL can be calculated by the equation given below:

DL = 3 × σ/S(2)

(where σ = standard error or deviation and S = slope of the calibration curve).

**Scheme 2:**
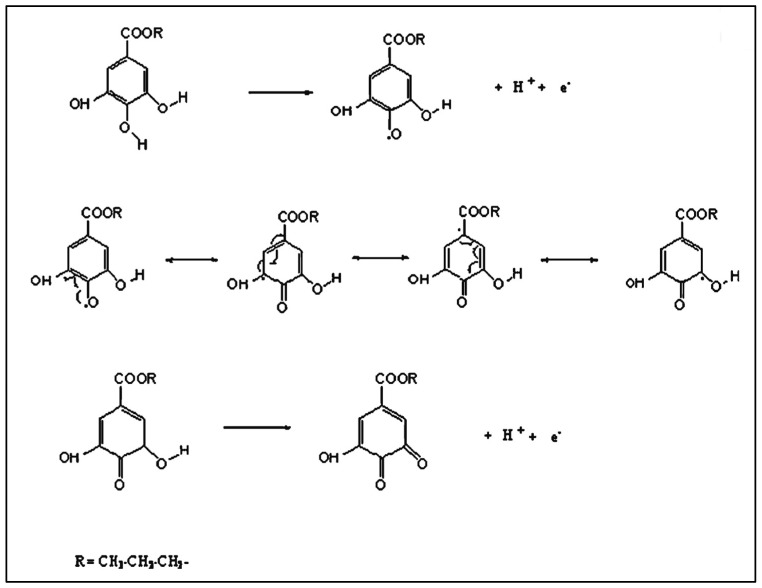
Reaction mechanism for PG detection. Reproduced with permission [21].

The improved performance may be attributed to the high surface area of the MWCNT-modified Au electrode. The authors also investigated the quantification of PG in vegetable oil samples, and the obtained results showed an acceptable recovery rate. The recovery rate of the proposed sensor was also validated by employing the spectrophotometric method. There was insignificant variation in the recovery rate. Thus, it may be considered that MWCNT-modified Au electrodes may be applied for real-time monitoring of PG in food samples. The probable reaction mechanism for PG detection is demonstrated in Scheme 2.

**Scheme 3:**
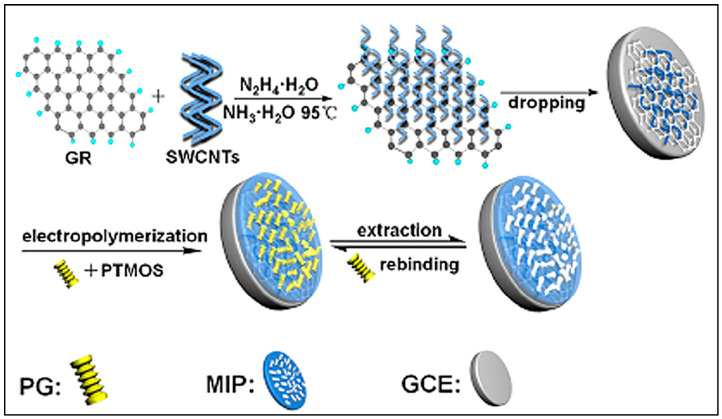
Schematic illustration of the fabrication of MIPs/Gr/SWCNTs/GCEs for PG detection. Reproduced with permission [22].

It can be observed in Scheme 2 that the oxidation of the hydroxyl group of the PG takes place during its quantification. An alkyl gallate radical is formed, which is then converted into the quinone, and the electrochemical reaction involves a 2e^−^, 2H^+^ transfer process. We believe that the DL of the reported electrochemical sensor should be further enhanced. In addition, despite excellent real-sample results, the above-mentioned sensor has yet to be explored for practical applications. Xu et al. [22] proposed the fabrication of a novel molecularly imprinted sol–gel electrochemical sensor for PG detection. The fabrication of the MIP-based sensor is illustrated in Scheme 3.

**Figure 2:**
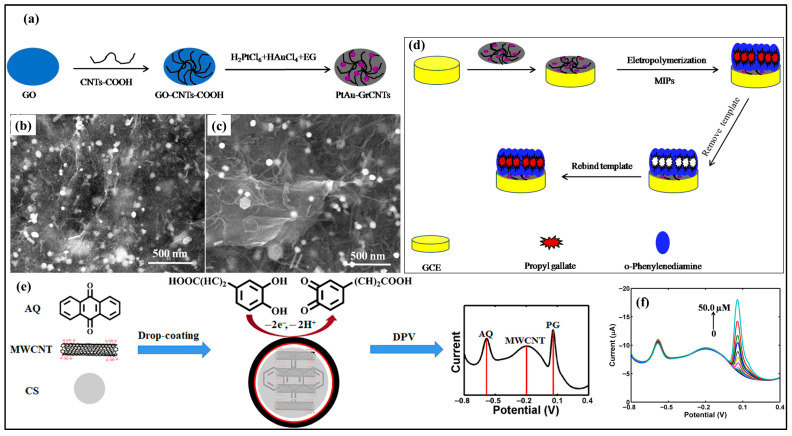
(**a**) A schematic representation showing the fabrication of the Pt-Au/GrCNTs composite. SEM image of (**b**) Pt-Au/Gr composite and (**c**) Pt-Au/GrCNTs composite. (**d**) A schematic illustration of the fabrication of an MIP sensor. (**e**) A schematic diagram showing the fabrication of an AQ/MWCNTs/CS/GCE-based dual-ratiometric sensor. (**f**) DPV curves of the AQ/MWCNTs/CS/GCE in the presence of various concentrations of PG. Reproduced with permission [23,24].

The glassy carbon electrode (GCE) was modified with a graphene (Gr)/SWCNT composite, and the sensing performance of the proposed MIP sensor was evaluated using cyclic voltammetry (CV) and DPV techniques. The proposed MIP sensor exhibited an interesting DL of 50 nM for PG detection. The authors also observed that the prepared electrode has the potential for the real-time detection of PG in noodles, cookies, and edible oil samples. Cui et al. [23] reported the fabrication of platinum (Pt)–Au-integrated Gr/CNT composites (Figure 2a). The synthesized materials were characterized by scanning electron microscopy (SEM) to evaluate their morphological features. The SEM image of the Pt-Au/Gr composite displayed the presence of Pt-Au nanoparticles (NPs) on Gr sheets (Figure 2b). The SEM image of the Pt-Au/GrCNTs confirmed the presence of Pt-Au/Gr with CNTs and authenticated the formation of the Pt-Au/Gr composite and Pt-Au/GrCNT composite (Figure 2c). The MIP sensor was fabricated through the electro-polymerization of an o-phenylenediamine film in the presence of template molecules on a GCE surface modified with Gr/CNT composites capped with Pt-Au bimetallic NPs, as shown in Figure 2d. The fabricated electrode displayed an enhanced surface area and electron transfer kinetics. The authors propose that the presence of the Pt-Au bimetallic NPs improved the surface area and catalytic activity of the fabricated electrode for PG detection. Thus, electrochemical studies displayed that enhanced electron transport kinetics and synergistic interactions between the prepared composites improved the detection of PG in terms of DL, sensitivity, and real-time detection in food samples. The study reported promising results for PG detection in 2015, but this could not be commercialized at the time. The researchers should focus on the commercialization of such sensors for practical applications. Yin et al. [24] reported the construction of a dual-ratiometric electrochemical sensor using an anthraquinone (AQ)/MWCNT/chitosan (CS) composite as sensing material (Figure 2e). The authors observed that current responses increase with increasing PG concentration, as shown in Figure 2f. The modified electrode AQ/MWCNT/CS/GCE (GCE = glassy carbon electrode) shows excellent performance for PG detection in terms of real-sample investigations in edible oil samples. The electrochemical investigations revealed that the sensing of PG involved a reversible redox reaction at the surface of the modified electrode, as shown in Figure 2e. The PG was electro-oxidized into quinone with the transfer of two electrons and two protons.

In another study, Albayati et al. [25] also developed an MIP-based electrochemical sensor for the monitoring of PG in mayonnaise and edible oil samples. The sensor was developed by the electro-polymerization of o-PDA on a MWCNTs/Au NP-modified GCE. The proposed MIP/Au NPs/MWCNTs/GCE sensor exhibited a DL of 6 nM and a wide LR of 0.01 to 5 µM and 5 to 1000 µM for the monitoring of PG. In addition, the authors found that the fabricated sensor has reasonably good reproducibility, selectivity, stability, and repeatability for PG detection. The authors also reported that the MIP/Au NPs/MWCNTs/GCE has an excellent recovery rate of 98.44% to 101.37% for PG detection in edible oils and mayonnaise samples. Despite such real-sample recovery rates of PG in edible oils and mayonnaise samples, the performance of the MIP/Au NPs/MWCNTs/GCE sensor is still limited to laboratory scale. Shi et al. [26] also used GO/β-cyclodextrin (GO/β-CD) as an electrochemical sensing material and applied it to the monitoring of PG in food samples such as edible oil. The electrochemical studies revealed that the synergism and catalytic activity of the GO/β-CD-modified GCE improved the DL for PG to 0.01 µM and displayed good electrochemical performance in the monitoring of PG in food samples. The GO/β-CD-modified GCE also displayed excellent selectivity for PG detection in the presence of various interfering substances, such as Cl^−^, Na^+^, K^+^, NO_3_^−^, glucose, Zn^2+^, Ca^2+^, citric acid (CA), Mg^2+^, Al^3+^, vitamin C, tartaric acid (TA), tert-butylhydroquinone (TBHQ), and gallic acid (GA). In another previous work [27], polystyrene sulfonate and oxidized MWCNTs (PSS/MWCNTs-COOH) were also deposited on an SPCE surface for the determination of PG. The electrochemical sensing performance of the fabricated electrode (PSS/MWCNTs-COOH/SPCE) was evaluated by employing an amperometric (Amp) method for the quantification of PG. The PSS/MWCNTs-COOH-modified SPCE delivered decent performance in terms of the DL (0.11 mg/L), repeatability, reproducibility, and satisfactory real-sample detection (recovery rate = 83–109%) in cosmetic samples. Although the above-mentioned studies clearly show that carbon-based and MIP-modified electrodes can enhance the PG detection by offering a higher surface area and efficient electron transfer, it is also observed that most of the reported advances are still limited to laboratory conditions. Even with low DL values and good recovery rates for the detection of PG in food or cosmetic samples, key practical aspects, such as scalable fabrication, long-term stability, etc., remain challenges for the scientific community. Therefore, practical application and commercialization of such PG sensors have not been explored.

### 2.2. Metal Oxide-Based Materials

**Figure 3:**
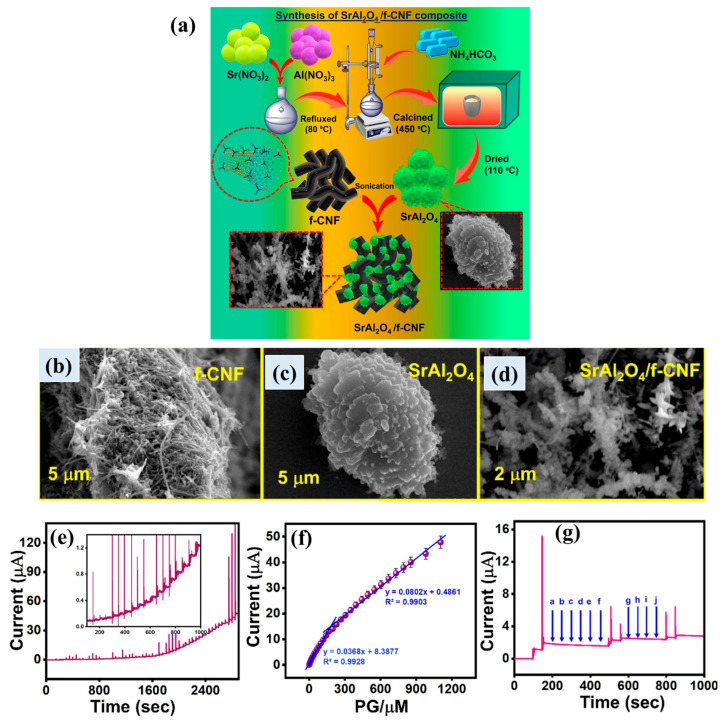
(**a**) Schematic graph shows the preparation of SrAl_2_O_4_/f-CNF. SEM image of (**b**) f-CNF, (**c**) SrAl_2_O_4_, and (**d**) SrAl_2_O_4_/f-CNF composite. (**e**) Amp data of SrAl_2_O_4_/f-CNF@RDE (inset shows enlarged data) and corresponding (**f**) calibration plot between current response and concentration of PG. (**g**) Selectivity test for PG detection. (a) Ascorbic acid (AA), (b) nitrofurantoin (NFT), (c) hydrogen peroxide, (d) sodium nitrite, (e) mercury ions, (f) enrofloxacin, (g) norfloxacin, (h) 2-nitroflourene, (i) flufenamic acid (FFA), and (j) chloramphenicol (CAP). Reproduced with permission [28].

Metal oxides are stable materials with reasonable catalytic activity, thermal stability, and cost-effectiveness. Therefore, it would be of great significance to employ metal oxides or oxide-based hybrid materials as sensing materials for the development of next-generation electrochemical sensors. Based on this, a synchronously activated strontium aluminate (SrAl_2_O_4_) nanoflake-anchored functionalized-carbon nanofiber composite was prepared using simple strategies for the construction of a PG sensor [28]. The SrAl_2_O_4_/f-CNF composite was prepared using reflux-assisted synthetic approaches, as shown in Figure 3a. The surface morphology of the SrAl_2_O_4_/f-CNF composite was checked by employing SEM. The observations revealed that f-CNF has highly entangled and closely packed fibrils, which may be due to the presence of hydrogen bonding and electrostatic interactions between the oxygen functionalities (Figure 3b). The SEM picture of SrAl_2_O_4_ is displayed in Figure 3c, which indicates the presence of a single cluster-like structure that is composed of tiny, flaky segments. The SrAl_2_O_4_ with a flaky structure was orderly aligned on the surface of CNF fibrils and formed the unique surface structure (Figure 3d). The X-Ray diffraction (XRD) pattern also exhibited the formation of an SrAl_2_O_4_/f-CNF composite with high phase purity. Furthermore, the authors utilized a SrAl_2_O_4_/f-CNF as an electrode modifier, which exhibited improved electron transfer by forming heterojunctions and enhanced the electrochemical sensing mechanism for PG. The Amp technique revealed that the SrAl_2_O_4_/f-CNF-based electrode shows a fast response for PG detection, as shown in Figure 3e. The current response linearly increases with respect to the concentration of the PG, as shown in Figure 3f. A broad LR of 0.1 to 1104.75 µM, DL of 0.075 µM, and sensitivity of 1.142 µA·µM^−1^·cm^−2^ were obtained under the optimized conditions. This sensor was also highly selective for the determination of PG in the presence of various interfering agents (Figure 3g). Although the SrAl_2_O_4_/f-CNF composite displayed efficient electron transfer and interesting sensing performance for PG detection, the reported findings were limited to the laboratory scale and could not be explored beyond laboratory conditions.

**Figure 4:**
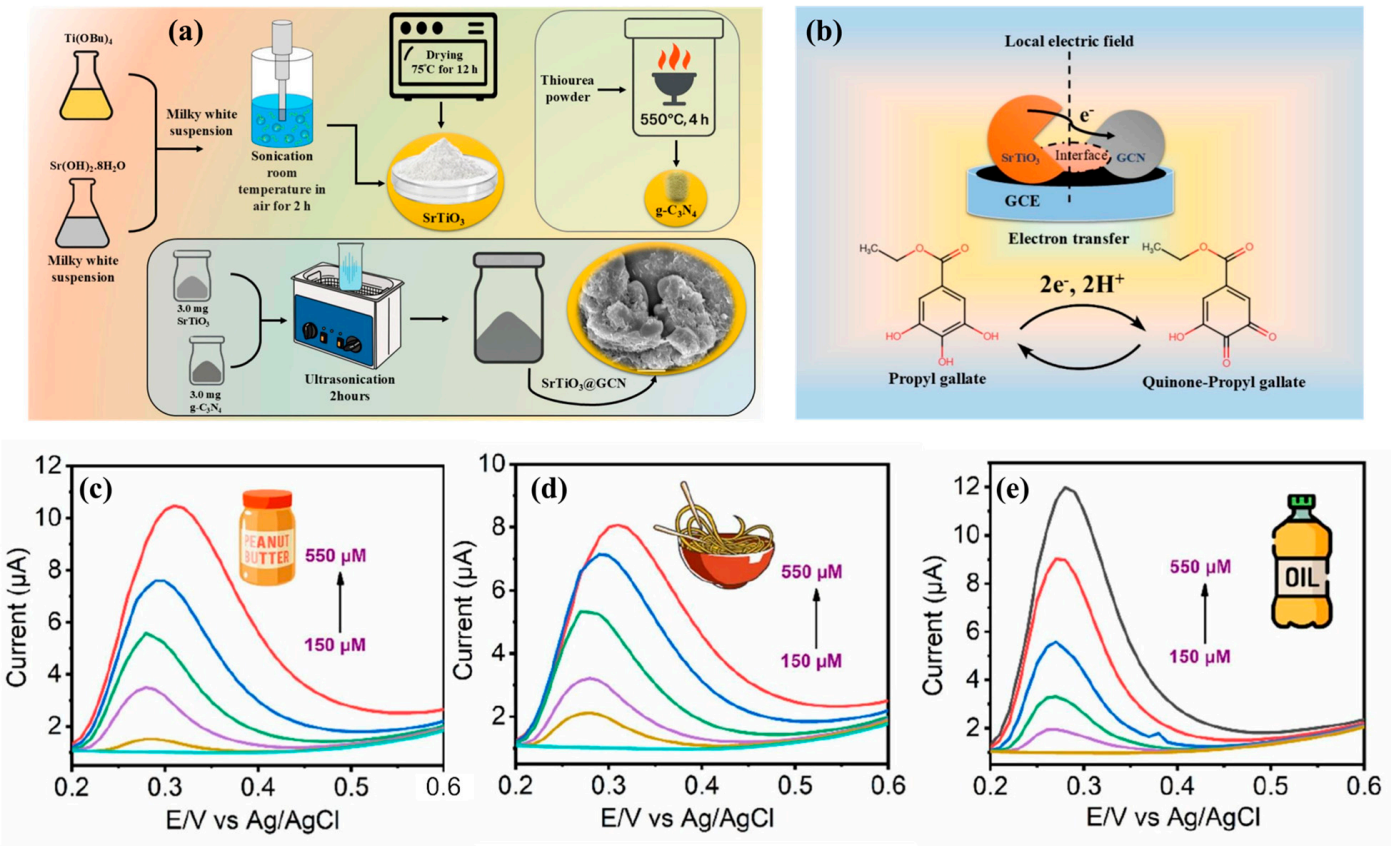
(**a**) Schematic diagram showing preparation of SrTiO_3_/gCN composite. (**b**) Schematic representation of electro-oxidation of PG at SrTiO_3_/gCN composite-modified electrode. DPV curves of SrTiO_3_/gCN composite-modified electrode for PG detection in (**c**) peanut butter, (**d**) noodle, and (**e**) oil samples. Reproduced with permission [31].

Chinnapaiyan et al. [29] reported that laser-induced graphene (LIG) offers several advantages, such as low cost and flexibility, for the construction of electrochemical sensors. The integration of LIG with metal oxide materials may enhance the detection of PG. In this regard, nickel–iron (NiFe) oxide was incorporated with LIG for the construction of an electrochemical sensor for PG detection. The fabricated porous NiFe oxide@LIG electrode displayed improved active sites, high conductivity, and electron transfer kinetics compared to the bare LIG electrode. The NiFe oxide@LIG electrode had a DL of 0.01 μM, LR of 0.5 to 5 μM, stability of 10 days, and sensitivity of 17.12 µA·µM^−1^·cm^−2^ for PG detection. The authors also found that the NiFe oxide@LIG electrode exhibited an excellent recovery rate of 79.2% for PG quantification. Despite the high sensitivity and smartphone integration, the above-mentioned study offers limited insight into long-term stability, large-scale fabrication process, and potential selectivity in real samples, which may hinder the practical application of the proposed NiFe oxide@LIG-based PG sensor. In another previous research article, Au-decorated iron oxide (Fe_2_O_3_) was also fabricated for the sensitive and selective detection of PG [30]. It is clear that Fe_2_O_3_ has several advantages, such as biocompatibility and higher electro-catalytic activity, and the presence of Au NPs enhances the conductivity of the resulting electrode material for the determination of PG. The Au@Fe_2_O_3_-modified screen-printed electrode (SPE) exhibited a DL of 10.3 nM and a wide LR of 0.16 μM to 500 μM, with excellent selectivity in the presence of various interfering substances. Despite its strong electrochemical sensing performance, the above-mentioned study does not clearly address the long-term stability of the Au@Fe_2_O_3_-based PG sensor. In addition, it does not address how easily it could be scaled up for practical or real-world applications. The sonochemically assisted formation of a strontium titanate (SrTiO_3_)-modified gCN composite was also reported by Gopakumar et al. [31], as shown in Figure 4a. This study focused on the detection of antioxidants, including PG. The mechanism for the electro-oxidation of PG at the fabricated electrode surface is displayed in Figure 4b. The SrTiO_3_/gCN composite was used as a sensing material for the determination of PG, and the authors achieved a decent DL of 0.014 μM and LR of 5.0 and 1300 μM using the DPV technique. The high reproducibility, selectivity (interfering substances = TBHQ, sucrose, Na^+^, KCl, glucose, and lactose), long-term stability, and satisfactory recovery of PG in peanut butter (Figure 4c), noodles (Figure 4d), and cooking oil (Figure 4e) samples suggest its potential for practical applications.

The enhanced performance of the SrTiO_3_/gCN composite-based electrode was attributed to the presence of synergism between the SrTiO_3_ and gCN. In another previous study [32], a zinc niobate NP-supported tomato-derived-biocarbon (ZnNb_2_O_6_/TBC) composite was fabricated using electrochemical approaches. The formation of the composite was confirmed by employing X-Ray photoelectron spectroscopy (XPS) and XRD analysis. The SEM results also authenticated the formation of the composite material with the presence of a layered sheet-like structure of TBC, which was attached with nanoflakes of ZnNb_2_O_6_. The ZnNb_2_O_6_/TBC was used as a sensing layer for the construction of the PG electrochemical sensor, and the CV study revealed that the ZnNb_2_O_6_/TBC-modified electrode had higher catalytic activity for the PG electro-oxidation process. The DPV analysis confirmed that the current response linearly increased with increasing PG concentration and delivered an LOD of 25 nM with a wide LR of 50 nM to 0.276 mM. The real-sample studies on biscuit samples also showed an LR of 50 μM to 850 μM, whereas a milk powder-based study exhibited an LR of 2 μM to 32 μM. This indicates that ZnNb_2_O_6_/TBC modified electrodes can be used for real-time monitoring of PG in food samples. Ye et al. [33] adopted a two-step hydrothermal method for the preparation of a novel bismuth vanadate-based Cu_3_(PO_4_)_2_/BiVO_4_ composite. This prepared Cu_3_(PO_4_)_2_/BiVO_4_ was coated on a GCE surface, and its electrochemical capability for PG detection was performed using the photoelectrochemical method. The fabricated sensor demonstrated an excellent DL of 0.005 nM for PG detection. The obtained results suggest that Cu_3_(PO_4_)_2_/BiVO_4_ has the potential to monitor the presence of PG in edible oil samples. As per the above-mentioned reports in Section 2.2, it can be noted that metal oxides and their hybrid material-based electrochemical sensors show decent sensitivity and wide linear detection ranges for PG quantification. This may be attributed to the improved electron transfer and synergistic interactions. However, most of the reported studies are still limited to laboratory conditions.

### 2.3. Selenides

Metal selenides and sulfides are promising catalytic materials for electrochemical applications due to their excellent physicochemical characteristics. Exploring this, a cobalt selenide (CoSe_2_) and iron sulfide (FeS_2_) composite was prepared through a hydrothermal method [34]. The study of the structural properties revealed that CoSe_2_ has a coral-shaped structure, whereas FeS_2_ has a sheet-like surface structure. The obtained composite exhibits better surface area, abundant active sites, and higher catalytic activity for PG detection. The CoSe_2_/FeS_2_-modified LIG electrode exhibited a DL of 0.018 μM, with decent sensitivity, high reproducibility, and selectivity for PG detection being observed under the optimized conditions. Although the CoSe_2_@FeS_2_ composite shows decent sensitivity and a low DL value for PG detection, the reported study was mainly limited to laboratory testing. Practical issues, such as long-term stability, consistency in large-scale production, and overall fabrication cost, were not clearly discussed. In future studies, such issues may be overcome to commercialize the potential of CoSe_2_@FeS_2_ composite-based PG sensors. Sakthivel et al. [35] also used a MoSe_2_/gCN composite as a sensing material for the monitoring of PG in food samples. The obtained results suggest that this PG sensor can be used for the monitoring of PG in real samples.

### 2.4. MOF-, LDH-, and MXene-Based Materials

**Figure 5:**
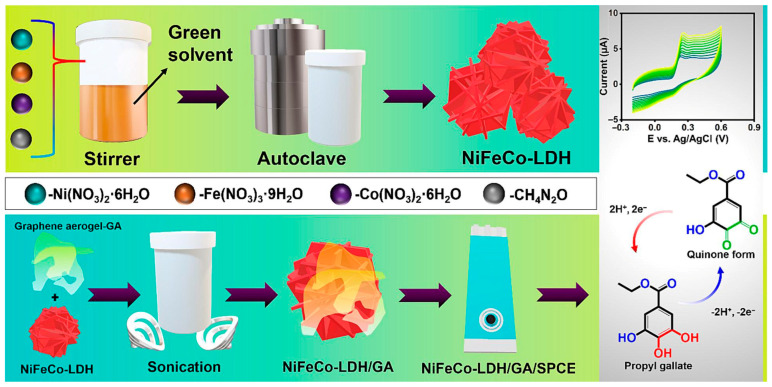
Schematic illustration of material preparation, electrode fabrication, and activity for PG detection. Reproduced with permission [38].

Metal–organic framework (MOF)-based materials offer several advantages, such as a high specific surface area, porosity, and active sites that may facilitate the charge transport kinetics of the electrochemical sensors. Given this, a cerium (Ce)–cobalt (Co) bimetallic–organic framework (CeCo-BMOF) was also investigated as a sensing material for the determination of PG [36]. The EIS study revealed the presence of decent electrical conductivity, which may facilitate electron transfer and enhance the detection of PG. A DL of 0.49 µM and wide LR of 1.5 µM to 17.6 µM and 11.5 µM to 458 µM were obtained for PG detection. This sensor also displayed good selectivity, reproducibility, and stability. The satisfactory recovery of PG in corn oil and peanut samples suggested its potential for real-time monitoring of PG in food samples. Srinivasan et al. [37] reported the preparation of a samarium (Sm)-based MOF and combined it with graphitic carbon nitride (gCN) to form a hybrid composite for the monitoring of PG. The obtained Sm-MOF@gCN composite-modified electrode delivered a DL of 2.05 nM and LR of 0.02 to 60 µM, with an excellent sensitivity of 0.1914 μA·μM^−1^·cm^−2^. The improved performance of the fabricated PG sensor was attributed to the bar shape of the Sm-MOF and the porous, sheet-like structure of gCN. The real-sample studies in cookies, noodles, cakes, and oils exhibited satisfactory recovery, which suggests its potential for practical applications. The layered double hydroxide (LDH) materials are the next-generation electrode materials for electrochemical applications. In connection with this, a nickel–iron–cobalt (NiFeCo) LDH was combined with a graphene aerogel (GA) in the construction of a PG electrochemical sensor as illustrated in Figure 5 [38].

The authors found that the 3D-3D heterojunction in the prepared composite enhances the electrochemical performance of the constructed electrode. The solvothermally prepared NiFeCo LDH/GA composite-modified electrode demonstrated a DL of 0.87 nM, sensitivity of 41.22 µA·µM^−1^·cm^−2,^ and LR of 0.001 μM to 297.43 μM for PG sensing. Stanley et al. [39] also explored a NiFeCu-LDH/GA/SPCE for PG detection. It was observed that the presence of a NiFeCu MOF with a porous GA provides a larger surface area and abundant active sites, which improve the determination of PG. The fabricated NiFeCu-LDH/GA/SPCE also displayed decent recovery of PG in food samples. In conclusion, MOF- and LDH-based composites show strong potential for PG sensing, particularly when combined with conductive carbon materials.

MXenes have received enormous attention because of their excellent conductive, layered, and catalytic properties. Previously, Parasuraman et al. [10] explored the potential of MXene-based materials for PG detection. In the study, a silver (Ag)-decorated titanium carbide (Ti_3_C_2_T_x_) MXene/cadmium telluride quantum dot (CdTe QD) composite was fabricated using hydrothermally assisted approaches. The Ag-Ti_3_C_2_T_x_/CdTe QD composite was coated on a GCE surface for the determination of PG. The authors observed that Ag-Ti_3_C_2_T_x_/CdTe QDs exhibit a high surface area, decent conductivity, and active sites that may facilitate electron transportation during the electrochemical oxidation of PG. The obtained results displayed a DL of 3.2 nM and sensitivity of 15.92 μA·μM^−1^·cm^−2^ for PG detection. In addition, the authors found that Ag-Ti_3_C_2_T_x_/CdTe QD-modified GCE may be explored for the detection of PG in chicken meat and ice cream samples. It is worth mentioning that CeCo-BMOF offers excellent selectivity and practical applicability for PG detection, but its sensitivity was found to be moderate compared to more advanced hybrid materials. The incorporation of gCN or graphene aerogels significantly enhanced the DL and sensitivity by improving the surface area and charge transfer. In contrast, LDH/Gr composites, especially those forming 3D heterojunctions, displayed reasonable analytical performance with low DL values. In addition, it was also found that most studies provide limited mechanistic insight and lack comprehensive comparisons with existing electrochemical sensors for PG detection, leaving questions about the long-term stability and real-world scalability.

### 2.5. Other Composite Materials

In another study [40], a novel PG electrochemical sensor was also fabricated using poly γ-aminobutyric acid (poly-GABA)/Au film as the sensing material. The Au had a needle-like, nanocluster-shaped morphology, which may enhance electron transfer during electrochemical reactions. The fabricated electrode demonstrated a DL of 0.001 µM and LR of 0.06 µM to 3.6 µM. The authors also found that the fabricated sensor had excellent recovery of PG in noodles, edible oils, and biscuits.

In another recent study [41], a molybdenum carbide (Mo_2_C)-anchored benzimidazole-intercalated functionalized-GO (fGO) material was prepared using simple synthetic protocols. The Mo_2_C@fGO-based electrode was used as a working electrode for PG detection and delivered a DL of 0.457 µM, an LR of 1 to 750 µM, decent selectivity, and reasonable recovery in packaged snacks and edible oil samples. The AuNPs/poly(p-aminobenzenesulfonic acid) [poly(*p*-ABSA)] composite was also adopted as an electrode material in the construction of a PG sensor [42]. The Au NPs/poly (*p*-ABSA)/GCE displayed a DL of 190 nM and excellent reproducibility and selectivity, with decent detection of PG in an edible vegetable oil sample. It is clear that the higher electrical conductivity and catalytic behavior of the prepared composite material enhanced the sensitivity of the proposed PG electrochemical sensor. For practical applications, electrochemical sensors should be highly selective, reproducible, stable, and sensitive with real-time monitoring of the targeted analyte. The selectivity of the determination of PG primarily arises from the synergistic interaction between the physicochemical properties of the electrode materials and the molecular structure of PG. It can be noted that PG contains phenolic hydroxyl groups and an aromatic ring, which may enable strong interactions such as hydrogen bonding, π-π interactions, and coordination with metal centers present in metal oxides, metal NPs, or hybrid inorganic composites. These interactions may promote preferential adsorption of PG on the electrode surface compared to the structurally dissimilar interfering species. Moreover, many inorganic materials (e.g., metal oxides, doped nanostructures, and composites) exhibit high electro-catalytic activity towards the electro-oxidation of PG. The high surface area, tunable surface chemistry, and abundant active sites of the electrode materials may facilitate faster electron transfer kinetics and lower oxidation over-potentials for PG detection. Thus, the sensitivity and selectivity of the PG sensors can be improved. In some cases, surface defects, oxygen vacancies, or specific crystal facets may further contribute to the selective detection of PG. It can be concluded that the above-summarized PG electrochemical sensors show decent sensitivity and real-sample recoveries. The electrochemical performance of the PG sensors may largely depend on the conductivity, catalytic activity, porosity, surface area, and electron transfer properties of the electrode materials. The electrochemical performance of the reported PG electrochemical sensors has been summarized in Table 1.

## 3. Fluorescence Sensors

It is worth mentioning that fluorescence-based detection of PG has also attracted the scientific community due to its advantages, such as a simple working principle, good selectivity, and high sensitivity. In recent years, numerous fluorescence sensors have been developed for the determination of environmental pollutants and food quality monitoring applications [43,44,45]. Fluorescence sensors have also been used for the determination of PG.

### 3.1. Selenides-Based Materials

It is understood that quantum dots (QDs) are semiconductor materials with a typical size of 2 to 10 nm. QDs exhibit excellent fluorescence and optical properties that make them a promising optical and fluorescence sensing material for the determination of the targeted analytes. Connected to this, Dwiecki et al. [46] reported the preparation of a cadmium selenide-modified zinc sulfide QDs (CdSe/ZnS QDs) composite. The synthesized CdSe/ZnS QDs were coated with β-CD and adopted as a sensing probe for the monitoring of PG in oils. The authors obtained a DL of 63 µM and a quantification limit (QL) of 184 µM. The CdSe/ZnS QDs also have excellent selectivity for PG detection in the presence of various interfering species such as GA, glucose, sinapic acid, methanol, and albumin. The fluorescence method involves a quenching-based mechanism for the detection of PG. Although this study demonstrated the sensing properties of the CdSe/ZnS QDs for PG detection through a fluorescence method, in-depth mechanistic aspects were missing. Future studies may consider it and investigate the in-depth mechanism for PG detection.

### 3.2. Carbon-Based Materials

**Figure 6:**
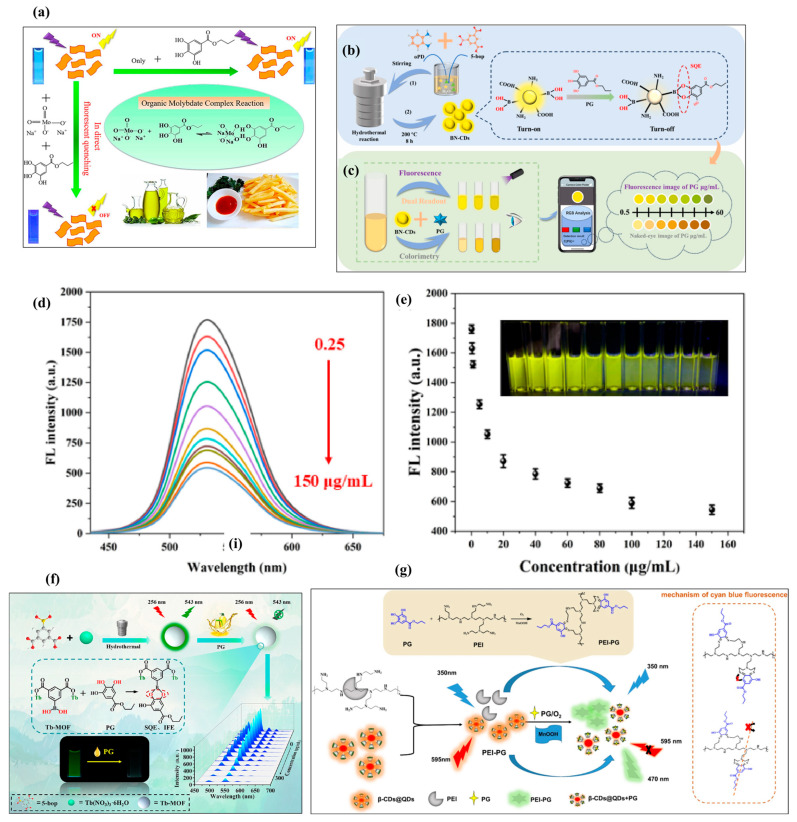
(**a**) Sensing mechanism for PG detection using organic molybdate complex [47]. Schematic graph shows preparation of (**b**) BN-CDs for PG detection and (**c**) dual-mode detection of PG. (**d**) Fluorescence data of BN-CDs probe with various concentrations of PG (0 to 150 μg/mL) and (**e**) related measured data. Inset shows images of various concentrations of PG [48]. (**f**) Schematic representation of sensing mechanism for PG using Tb-MOF [49]. (**g**) Schematic picture for PG detection mechanism [52]. Reproduced with permission [47,48,49,52].

Carbon-based materials exhibit excellent conductive and surface properties. Carbon-based materials are also utilized as a template for the synthesis of other materials. Yue et al. [47] proposed the fabrication of a novel fluorescence PG sensor by utilizing the quenching properties of an organic molybdate complex (OMC), which is formed in the reaction of MoO_4_^2−^ and PG to gCN, as shown in Figure 6a. It can be seen that PG reacts with molybdate to form the organic molybdate complex. This formed organic molybdate complex quenches the fluorescence of gCN. As the concentration of PG increases, more organic complexes of molybdate are formed, resulting in a greater quenching effect. The proposed fluorescence sensor delivered a DL of 0.11 μg/mL with satisfactory recoveries. This method was found to be a rapid process for the detection of PG. Xu et al. [48] also adopted a hydrothermal method for the preparation of boron (B)–nitrogen (N) co-doped carbon dots (BN-CDs), as shown in Figure 6b. The synthesized composite was used for the determination of PG in food samples, and the sensing process for dual-mode PG detection has been described in Figure 6c. Figure 6d shows the fluorescence data for PG detection in the presence of various concentrations of PG, and the related measured data is summarized in Figure 6e. These observations revealed that BN-CDs are a promising material for the monitoring of PG through the fluorescence method.

### 3.3. MOF-Based Materials

In another study [49], a boric acid-based Tb-MOF system was prepared in facile conditions. The Tb-MOF (using 5-boronoisophthalic acid (5-bop) as ligand) exhibited multiple emissions at 490, 543, 585, and 622 nm under the excitation wavelength of 256 nm. The authors also observed that the fluorescence of the Tb-MOF was significantly weakened in the presence of PG due to the nucleophilic reaction of the boric acid of the Tb-MOF and the o-diphenol hydroxyl of the PG, as well as static quenching and internal filtering. The rapid response of the proposed sensor for PG detection and the interesting LR of 1 to 150 μg/mL and DL of 0.098 μg/m suggested its potential for the determination of PG in food samples such as soybean oil. The mechanism for PG detection has been illustrated in Figure 6f.

### 3.4. Ruthenium (Ru) Complex and Materials

In other previous investigations, it was found that the boric acid group–functional-Ru complex has excellent fluorescence properties for the monitoring of PG [50]. Therefore, the proposed Ru complex-based material was used as a fluorescence probe for the monitoring of PG, and the authors achieved a DL of 0.26 µM. The authors stated that, when excited at 480 nm, the probe displayed a strong emission peak at 620 nm, which was specifically quenched by PG at a pH value of 7.0, owing to the covalent interactions between the probe’s boric acid group and the o-diphenol hydroxyl groups of PG. Liu et al. [51] also used a Ce(IV)-coordinated organogel-based assay for the determination of PG using turn-on fluorescence signal-based strategies. The authors synthesized a Ce^4+^-coordinated organogel using simple strategies. It was observed that Ce^4+^ triggered a fluorogenic reaction between the PG and PEI (PEI = polyethyleneimine). The proposed turn-on fluorescence sensing probe demonstrated a DL of 1 μg/mL and acceptable recovery of 80.22% to 106.2% in surface water and edible oil (peanut oil and almond oil) samples. It was also stated that functionalized-organogel-based sensors can be combined with smartphones for environmental health and food safety applications. Peng et al. [52] proposed the fabrication of MnOOH nanoflake/β-cyclodextrin modified quantum dots (QDs) for PG detection. The synthesized β-CDs@QDs exhibited excellent sensing behavior for the monitoring of PG. The mechanism for the determination of PG has been depicted in Figure 6g. The obtained results demonstrated an LR of 0.1 to 35 μg/mL, DL of 0.023 μg/mL, and QL of 0.077 μg/mL for PG detection. The performance of the fluorescence sensors for PG detection has been summarized in Table 2.

## 4. Conclusions and Future Perspectives

Finally, to conclude, the present review article summarizes the previously reported literature on the electrochemical and fluorescence detection of PG. As per the summarized reports, it has been found that the electrochemical method has significant advantages for the determination of PG using composite materials. Various electrode materials such as metal oxides, MOFs, carbon-based materials, polymers, LDHs, etc., have been explored for the electrochemical detection of PG. The electrochemical methods provide low cost, simplicity, high selectivity, and portable detection. The reported performance for PG detection was found to be promising and displayed potential for commercialization. However, the long-term stability, real-time monitoring of PG in the presence of interfering substances, and scalability issues need to be overcome. We believe that MXene-based materials and LDH composites are the next-generation electrode materials for the effective detection of PG. Future research may focus on the development of PG electrochemical sensors using MXene- and LDH-based hybrid composite materials. However, eco-friendly synthesis methods for MXene preparation should be developed. In contrast, the fluorescence method also offers high sensitivity, low detection limit, and fast response for PG detection. The fluorescence method can be used for the monitoring of PG in real samples, including edible oils. It can be stated that fluorescence has several advantages for laboratory-based, ultra-sensitive analysis in food samples, whereas electrochemical methods are often considered superior for practical on-site monitoring of PG. Moreover, only a few reports are available on the development of fluorescence-based detection of PG. The depth of the mechanistic aspects should be researched, and more studies should be conducted on the fluorescence-based detection of PG.

## Figures and Tables

**Table 1:** Electrochemical performance of the previously reported PG electrochemical sensors.

Material	DL	LR	Real Sample	Sensing Technique	References
MWCNTs	0.63 µM	10 to 100 µM	Vegetable oils	DPV	[21]
Gr/SWCNTs	0.05 µM	0.08 to 2600 µM	Noodles, edible oils, and cookies	DPV	[22]
Pt-Au/GrCNTs	0.0251 µM	0.07 to 10 µM	Vegetable oil	CV/Amp	[23]
AQ/MWCNTs/CS/GCE	0.67 µM	1 to 30 µM	Edible oil	DPV	[24]
MIP/GNP/MWCNTs	0.006 µM	0.01 to 5 and 5 to 1000 µM	Soybean oil, black cumin oil, and mayonnaise	DPV	[25]
NH_2_-β-CD/rGO/GCE	0.01 µM	0.1 to 30 µM	Edible oil	DPV	[26]
PSS/MWCNTs-COOH/SPCE	0.11 mg/L	0.37 mg/L to 10 mg/L	Cosmetics	Amp	[27]
SrAl_2_O_4_/f-CNF@GCE	0.075 µM	0.1 to 1104.75 µM	Canola oil, olive oil, and meat	Amp	[28]
NiFe-oxide@LIG	0.01 µM	0.5 to 5 µM	Edible oil, chicken extract, and cookies	DPV	[29]
Au@Fe_2_O_3_/SPCE	0.0103 µM	0.16 to 500 µM	Noodles and cookies	DPV	[30]
SrTiO_3_@gCN	0.014 µM	5 to 1300	Peanut butter, cooking oil, and noodles	DPV	[31]
ZnNb_2_O_6_@TBC	0.025 µM	0.050 to 276 µM	Biscuit and milk powder	DPV	[32]
CoSe_2_@FeS_2_	0.018 µM	0.05 to 2000 µM	Oil and chicken	DPV	[34]
MoSe_2_@g-C_3_N_4_	0.073 µM	0.1 to 1285	Coffee cream, edible oil, and ice cream,	DPV	[35]
CeCo-BMOF	0.49 µM	1.5 to 458 µM	Peanut oil and corn oil.	DPV	[36]
Sm-MOF@GCN	0.00205 µM	0.02 to 60 µM	Cookies, cakes, oils, and noodles	DPV	[37]
NiFeCo-LDH/GA	0.00087 µM	0.001 to 297.43 µM	Oil and cookies	DPV	[38]
NiFeCu-LDH/GA	0.004	0.02 to 279.1 µM		DPV	[39]
AuNC/pGABA	0.001 µM	0.06 to 3.6 µM	Biscuits, edible oils, and noodles	CV	[40]
Mo_2_C@fGO	0.457 µM	1 to 750 µM	Edible oils and packaged snacks	DPV	[41]

**Table 2:** Sensing performance for PG detection using fluorescence method.

Material	DL	LR (µM)	Real Sample	Sensing Technique	References
CdSe/ZnS	63 µM	5 to 148 µM	Oil	Fluorimetric	[46]
OMC	0.11 µg/mL	0.5 to 200 µg/mL	Edible oil	Fluorimetric	[47]
BN-CDs	0.12 µg/mL	-	Tap water, peanut, and corn oil	Fluorimetric	[48]
Tb-MOF	0.098 µg/mL	1 to 150 µg/mL	Soybean oil	Fluorimetric	[49]
Ru complex	0.26 µM	-	Soybean oil, sesame oil, and potato chips	Fluorescence	[50]
Ce(IV) coordinated organogel	1 mg/mL	-	Surface water, peanut oil, and almond oil	Fluorescence	[51]
β-CDs@QDs	0.026 µg/mL	0.1 to 35 µg/mL	Shrimp strip, potato chips, and biscuits	Ratio-fluorescence	[52]

## Data Availability

No new data were generated. The authors are unable to provide data.
